# Silent Screams: A Narrative Review of Cyberbullying Among Indian Adolescents

**DOI:** 10.7759/cureus.66292

**Published:** 2024-08-06

**Authors:** Vijayarani M, G Balamurugan, Sanjay Sevak, Kusum Gurung, Bhuvaneswari G, Sangeetha X, Thenmozhi P, Tamilselvi S

**Affiliations:** 1 Psychiatric Nursing, Employees' State Insurance Corporation (ESIC) College of Nursing, Bengaluru, IND; 2 Psychiatric Nursing, National Institute of Mental Health and Neurosciences (NIMHANS), Bengaluru, IND; 3 Mental Health Nursing, All India Institute of Medical Sciences, Jodhpur, IND; 4 Mental Health Nursing, Ramaiah Institute of Nursing Education and Research, Bengaluru, IND; 5 Community Health Nursing, Saveetha College of Nursing, Saveetha Institute of Medical and Technical Sciences, Chennai, IND; 6 Obstetrics and Gynaecological Nursing, Ramaiah Institute of Nursing Education and Research, Bengaluru, IND; 7 Medical Surgical Nursing, Saveetha College of Nursing, Saveetha Institute of Medical and Technical Sciences, Chennai, IND

**Keywords:** mental health, consequences, impact, prevalence, india, adolescents, victimization, cyber bullying

## Abstract

Cyberbullying is bullying with the use of digital technologies, which can take place on social media, messaging platforms, gaming platforms, and mobile phones. It is repeated behavior aimed at scaring, angering, or shaming those who are targeted. India happens to be one of the rapidly improving countries in the cyber world and thus faces a lot of problems regarding cyber crimes, especially cyberbullying.

This narrative review aims to provide a thorough assessment of the impact of cyberbullying among Indian adolescents. The database engines such as PubMed, Google Scholar, and PsycINFO were searched relevant to the Indian context, focused on cyberbullying and victimization among adolescents, and published within the last 10 years (2014-2024) were included. Around 19 articles were reviewed and analyzed.

Cyberbullying in India is on the rise due to increased technology access, social media, and insufficient awareness and prevention measures, with significant gender differences in aggression patterns. The severe psychological and physiological effects on victims, including depression and stress-related health issues, highlight the need for accurate data and culturally tailored interventions. Studies show varying prevalence rates, emphasizing the urgent need for focused efforts to combat cyberbullying among Indian youth. The review encompasses various aspects, including prevalence, standard methods, forms, causes, consequences, and effects on mental health factors contributing to cyberbullying in India. Additionally, the review explores cyberbullying during COVID-19 and interventions for cyberbullying and highlights the evidence from cohort studies, mixed-method studies, and systematic reviews.

A growing number of adolescents are experiencing cyberbullying, which has a severe impact on their lives and leads to unexpected deviances. Cyberbullying remains a growing threat, requiring stronger, coordinated action by the government to genuinely make a difference and safeguard adolescents in India.

## Introduction and background

Traditional bullying is "a psychosocial problem of intentionally and repeatedly harming others and creating an imbalance of power between the victim and the perpetrator, with negative consequences for both parties". With traditional bullying, most of the bullying was in terms of physical threats of a stronger person or group expressing their power over weaker people. Cyberbullying often shares similar, if not identical, definitions and generally involves an imbalance in social and psychological abilities. "Online bullying is defined as bullying over the Internet or by text message." Some examples of online bullying would be chat rooms, social networks, mobile applications, and electronic games. According to surveys, the rate of bullying increases every successive year, although parents commonly report that children are often bullied at school. Accordingly, 19.2% of children are bullied through social networks and online programs, while 7.9% of children are bullied through electronic video games [1].

Cyberbullying can be defined as harassing, defaming, or intimidating someone over the internet, generally using mobile phones or computers, on social media, online chat groups, or any other online platform. It includes actions intended to degrade, belittle, or harass another person, and it can have a long-lasting traumatic impact on the individual [2]. Cyber victimization has been defined as the aggressive behavior experienced while using the modes of information and communication this may include the internet, gaming consoles, or smartphones. In this way, individuals become victims of violence, harassment, trolling, stalking, bullying, and crimes in the cyber world [3]. India, a nation that is expanding quickly in cyberspace, is seeing a sharp rise in cybercrimes, including cyberbullying. India has the highest incidence of internet harassment, with over 33% of children reporting having been the victim of it. The number of cybercrimes increased by 63.48% in 2019 compared to 2018, per NCRB data. This problem highlights the dangers that strangers and impersonators offer, particularly to children and youth. Aggressive behaviors that form cyber victimization include harassing messages and emails, disparaging comments, posting or sharing humiliating pictures or videos, online threats, intimidation, and blackmail [4-9]. Cyberbullying may include public shaming or spreading false information. Cyberstalking, through which the person is followed, and his/her activities online are monitored, romance scams, and other kinds of financial fraud. Doxing is a process of revealing private information about a person without consent; phishing, relating to theft attempts on sensitive information like passwords or credit card numbers; identity theft and unauthorized access to personal accounts or data [4-9].

Cyberbullying and victimization among adolescents in India are a fast-growing concern that needs to be taken seriously. Among various effects, cyber victimization has led to a wide range of negative mental health outcomes, such as depressive symptoms, anxiety, distress, somatic health complaints, and self-harm [4-8]. Cyberbullying is deeply pervasive in developed countries. For instance, developed countries such as India and Brazil become the leading two nations for this infused activity of cyberbullying [4]. It has also been linked to more serious outcomes, such as cyber suicide, where youths may webcast their suicide or, using the internet, enter into suicide pacts. Experiences of being victimized online amplify distress and may exacerbate existing mental health disorders, leading to cognitive disturbances, impulsive behaviors, and memory difficulties [4]. The mental health impacts of cyber victimization are particularly poignant in the context of limited access to mental health care and remote support networks during the COVID-19 pandemic [3].

It is against this background that understanding the prevalence and consequences of cyberbullying and victimization among adolescents in India is imperative for development in the design of effective prevention and intervention strategies. Considering its adverse effect on mental health, the increasing prevalence of cyber victimization among adolescents in India calls for urgent attention and effective prevention and intervention [9]. It was established from the studies that the technological platforms most frequently misused for cyberbullying were instant messaging and chat rooms. Other media platforms where such harmful activities are noted to take place include social networking sites such as Facebook, Twitter, and MySpace [9]. Particular media forms that have been studied in terms of victimization from cyberbullying include mobile phone calls, text messages, emails, picture/video clip bullying, instant messaging, chat rooms, websites, and massively multiplayer online games [9].

Cyberbullying is an emerging issue, gaining prominence very fast as one of the greatest concerns across the world, particularly true for India. India, with its growing digital economy, has experienced a rapid rise in cybercrimes; amongst them, cyberbullying has emerged as the most alarming trend [2]. According to NCRB, in the year 2020, a total of 50,035 cases of cybercrime were reported in India, out of which 1614 cases of cyberstalking, 762 cases of cyber blackmailing, 84 cases of defamation, 247 cases of fake profiles, and 838 cases of fake news were investigated. As per the data provided by NCRB, cybercrimes in India increased by 63.48% from 2018 to 2019 from 27248 cases to 44548 cases, and further up surged by 12.32% in 2020 from 44548 cases to 50035 cases [2]. The rise in cases of cyberbullying in India is very complex and is fostered by several factors, including a lack of digital literacy, an unsatisfactory level of cyber safety awareness, and the absence of robust legal frameworks for handling the problem. The Indian government has already passed some legislation aimed at curbing cyberbullying, such as reporting portals, awareness campaigns, and legal provisions. That said, cyberbullying remains a growing threat, requiring stronger, coordinated action by the government to truly make a difference and safeguard the vulnerable segments of the population, especially children and adolescents [2].

Hence, the review of cyberbullying and victimization among adolescents in India is indispensable in the wake of its increasing prevalence and hazardous effects. A review of such literature could help one in crucial ways to comprehend the factors leading to cyberbullying, the characteristics associated with victims and perpetrators, the psychological effects among adolescents, and effectiveness of the existing measures and interventions. Besides, this would create awareness about the context-specific challenges faced by Indian adolescents vis-à-vis cyberbullying and victimization, taking into consideration the cultural, social, and technological factors. This review can help in giving a base for developing prevention and intervention strategies to minimize cyberbullying and protect the well-being of Indian adolescents. Thus, the current review will help us deeply understand the factors that drive this issue, the impact on adolescents, and the effectiveness of current measures. The objectives of this review are firstly to present an overview of the prevalence and impact of cyberbullying on adolescents in India, secondly to take stock of the efforts that have been undertaken so far by the government and other stakeholders, thirdly to identify the gaps existing in the strategies developed until now, and finally to propose holistic efforts to effectively reduce cyberbullying and create a safer online environment for adolescents of India. This review would provide policymakers, educators, parents, and other stakeholders insight into developing evidence-based strategies to prevent and address cyberbullying.

## Review

This review aims to assess the forms of cyberbullying, its prevalence, risk factors, psychological impact, and preventive strategies for cyberbullying among adolescents in India 

Methods

The methods for this narrative review of cyberbullying are as follows: This involved literature searches on PubMed, Google Scholar, and PsychINFO for publications from the last 10 years (2014-2024). The searched terms used were cyberbullying, cyber bullies, cyber victims, cyber victimization, online bullying, cyber aggression, cyber violence, electronic aggression, Internet harassment, victimization, adolescents, and India. Inclusion criteria for the present study were articles and studies specifically focused on cyberbullying among adolescents in India, research involving adolescents, defined as individuals aged 10-19 years, articles providing data on the prevalence of cyberbullying incidents and their impacts on Indian adolescents, studies detailing efforts by the Indian government to prevent and address cyberbullying among adolescents, and research focusing on the psychological and mental health consequences of cyberbullying on victims, including depression, anxiety, suicidal ideation, and other related issues. In the process, case studies, non-refereed, and non-English publications were excluded.

In total, 19 articles were reviewed, analyzed, and synthesized as following themes: (i) Prevalence and Forms of Cyberbullying; (ii) Factors Contributing to Cyberbullying; (iii) Consequences of Cyberbullying; (iv) Interventions and Coping Strategies; (v). Legal and Policy Framework.

Prevalence and Forms of Cyberbullying

Prevalence of cyberbullying in India: While no complete data exists on the prevalence of cyberbullying in India, what is available as information suggests that it is a major problem. Prevalence studies from India have shown that a high percentage of adolescents experience or engage in cyberbullying. The prevalence varies across different regions of the country. The prevalence of cyberbullying among adolescents in India varies according to different studies. A study conducted among 228 teenagers between the ages of 11 and 15 years reported a prevalence rate of 17.2% for cyberbullying victimization [10]. Another survey in Delhi with 174 middle graders reported 8% engaging in cyberbullying and 17% reporting victimization through such acts [11]. Comparing boys with girls, the results showed that boys were offenders of cyberbullying and also victims of cyberbullying, while only victims of cyberbullying were victimized by both boys and girls [10]. Another study involving 1,721 students from higher education institutions in India showed that 30% of the respondents experienced cyberbullying [2]. Overall, these findings tend to suggest that, although cyberbullying is an important issue among Indian adolescents, the exact rates can vary by location and the sample under study. A mixed-methods study conducted in the Kozhikode district of Kerala state, India, touched upon the aspect of cyberbullying among various types of bullying experiences by adolescents. In the study, 3.3% of the respondents reported experiencing cyberbullying [12]. The prevalence of cyberbullying among nursing students in India has been recognized as an issue of concern. In a study, the overall prevalence of cyberbullying was found to be alarming, standing at 42.8%, with males experiencing slightly higher rates than females. Additionally, 26.3% of participants reported significant adverse effects on their academic performance as a direct consequence of cyberbullying [13]. A study conducted by Gupta et al 2023 found that among 213 medical students, One hundred and twenty-nine students (60.56%) became victims of some form of cyberbullying. One hundred and twenty-four students (58.22%) indulged in cyberbullying other students. One hundred and three students (48.36%) were both bullies and victims. These figures indicate that cyberbullying is a major issue among nursing students in India, thus warranting further research with respect to its prevention and management. The prevalence of cyberbullying among Indian adolescents, as shown by the host of studies, brings to the fore the pressing need for attention and intervention.

According to NCRB, 50,035 cases of cybercrime were reported in India in the year 2020 [2]. This is an alarming trend and presents a serious need to act against threats posed by harassment, impersonation, and online abuse, which may turn out to be potentially disastrous to young victims. The existing research on cyberbullying in India has largely focused on school-aged children, and there is relatively little known about university students [8]. This is a critical gap, as higher education institutions also face serious challenges in dealing with the increasing incidences of digital harassment and victimization [8]. Thus, the prevalence of cyberbullying among adolescents in India ranges from 3.3 to 60.56%.

Forms of cyberbullying in India: Some of the kinds of cyberbullying in India include bullying through text messages, phone calls, emails, instant messengers, and social media; publishing hurtful words, derogatory comments, or fake information on public forums or blogs. Hacking accounts for personal vendetta; threats of rape or death; revenge porn, which is posting sexually explicit images or videos of a person online without consent; engaging in online polls to body-shame victims; forming groups to spread false rumors or share morphed pictures and videos [14,15]. These forms of cyberbullying in India highlight the range of tactics used by perpetrators to harass and harm their targets.

Common methods of cyberbullying among school-going adolescents: The menace of cyberbullying, due to the rise in social media and online activities, has affected the maximum percentage of Indian adolescents. Some of the typical ways of cyberbullying among school-going adolescents include rumor spreading, fun-making, sending hurtful messages, stalking, making insulting remarks, leaking and sharing photos, or videos through electronic media the internet [10], sharing confidential information with others without consent of the victim, name-calling, trolling, threatening to hurt, online harassment, death threats, and sharing sexually explicit offensive messages without consent (Table 1). Cyberbullying can involve using social networking sites such as Instagram, Facebook, Twitter, WhatsApp, Snapchat, YouTube, and live gaming among others, to perpetuate their acts (Table 2). Threats and embarrassing content can also be sent via text message, email, and the creation of fake accounts in order to target someone online [10]. The research on aggression in schools, cyberbullying, and gender issues established that out of 174 middle graders in Delhi, 8% practiced cyberbullying through hacking, circulating private photos and videos, and circulating fake or false information [11]. Other common forms of cyberbullying identified in the Indian context are online harassment, trolling, exclusion, and impersonation [11].The majority of people who were subjected to cyberbullying experienced more than one type of cyberbullying.

**Table 1:** Common methods of cyberbullying among adolescents in India

Common methods of cyberbullying
Stalking
Rumor spreading
Making insulting remarks
Fun-making
Sending hurtful messages
Leaking and sharing photos, or videos through electronic media
Sharing confidential information with others without the consent of the victim
Online harassment
Name-calling
Trolling
Threatening to hurt
Death threats
Sharing sexually explicit offensive messages without consent
Hacking
Circulating private photos and videos
Circulating fake or false information

**Table 2:** Common networking sites where cyberbullying happens among adolescents in India

Cyberbullying can involve using social networking sites such as
Instagram
Facebook
Twitter
Snapchat
YouTube
WhatsApp
Livegaming

Most common types of cyberbullying reported in the print media in India: According to the print media in India, some of the most common kinds of cyberbullying reported are harassment through social media platforms, spreading rumors and false information, blackmail and extortion, and online stalking. The most common types of cyberbullying reported in the print media were online harassment and impersonation [16]. In the print media, male victims of cyberbullying were reported in 56.9% (29 out of 51) of the articles, whereas female victims were reported in 27.5% (14 out of 51) of the articles. In 15.7% (8 out of 51) of the articles, the gender of the victims was not mentioned [16]. In the print media, 56.9% of victims were reported as males, while females made up 27.5% of the victims. The gender of the victims was not identified in 15.7% of the articles [16]. The majority of the individual stories on cyberbullying reported in the examined newspapers attributed cyberbullying to societal reasons. That is, most of the individual stories on cyberbullying reported in the sampled newspapers attributed societal reasons for the cause for cyberbullying.

A McAfee Corp report divulged that 85% percent of children in India have been a victim of cyberbullying, the highest in any of the countries surveyed. Nearly half, 45%, acknowledged cyberbullying a stranger, while at 48%, they confessed they cyberbullied someone they know. These figures, when compared to global averages, come in substantially higher-17% and 21%. Apart from this, 42% of children in India have faced racist cyberbullying, 36% claimed to have been trolled, and 30% claimed to have faced sexual harassment and threats of personal harm, which is twice as much as the global average. The girls in the age group of 10-16 years are also vulnerable, with 32-34% reporting sexual harassment and threats against them, a bit above the global average. Of course, where the global average was at 64%, more than 45% of Indian adolescents in the survey hid their cyberbullying experiences from parents, perhaps because of lesser conversation around it [17].

Cyberbullying during COVID-19: Within the COVID-19 pandemic in India, as elsewhere, online activities increase due to lockdowns and measures of social distancing, which then increase the risks of cyberbullying. These then have significant negative impacts on the mental health of individuals who are most susceptible to such bullying, especially those belonging to sexual minorities. The experience of cyberbullying puts one at risk of adverse emotional consequences, changed behavior, and potential psychopathologies. The specific impact on sexual minorities in India was verbal-aggressive behavior, sexual cyberbullying, and attacks on identity. These experiences have been said to elicit feelings of distress, depression, anxiety, and other mental health issues since individuals are most often targeted for their sexual orientation and identity [18]. In addition, the stress and anxiety that cyber victimization brings with it would result in somatic health complaints such as headaches, stomach pains, and a host of other stress-related somatic complaints [3]. This combination of mental and physical health challenges can lead to a seriously debilitated state of well-being for an individual [18].

Factors Contributing to Cyberbullying

Factors contributing to cyberbullying in India: The rise in cases of cyberbullying in India can be based on several reasons, including increased access to technology and social media and the lack of adequate awareness and prevention measures. Secondly, research has indicated that gender significantly impacts cyberbullying, where male students tend to engage in both online and offline aggression [10]. This points to the need for targeted interventions that address the unique challenges faced by different genders.

According to various pieces of research, some factors that are said to contribute to the rising instances of cyberbullying in India include increased usage of the internet, lack of parental monitoring, engagement with social media, and lack of awareness about online safety [2]. Cyberbullying can be enacted by adolescents whose offline lives are burdened by stress and power imbalance as a way of coping or revenge [19]. In addition, anonymity on the internet emboldens perpetrators, and they often don't consider the implications of their acts in the real world. Also, the lack of effective and established legal frameworks and meaningful enforcement procedures has facilitated cyberbullying in the country [2].

Causes and consequences of cyberbullying in India: The prevalence of cyberbullying in India could be attributed to factors that include the rapid proliferation of digital technologies, an extraordinary lack of digital literacy, and the absence of a robust regulatory regime. The anonymity provided by online platforms, coupled with the ease of access to social media and messaging apps, has emboldened perpetrators to engage in abusive and harmful behavior with little fear of consequences [2]. Therefore, one major impetus of cyberbullying in India is the digital literacy and awareness of children and adults. Young people and many users are not well educated on using responsible and ethical digital tools; hence, harmful online behavior is normalized. Moreover, the limited awareness of reporting mechanisms of cybercrime and provisions of the law intensifies the problem to the extent that victims often feel helpless and unprotected [10]. The impact of cyberbullying can be disastrous, especially for young victims. Many studies show that targeted children stand at higher risk of developing mental problems, including depression, anxiety, and low self-esteem. Some cases of cyberbullying have been associated with suicidal thoughts and suicide attempts, emphasizing the urgent need for effective intervention and support [4].

Motives of cyberbullying and victimization in India: Cyberbullying in India is a multi-faceted and complex issue, driven by various factors. The motives for cyberbullying among adolescents in India vary from a desire to derive pleasure by hurting the victim to feeling jealousy or revenge, seeking power and superiority, enhancing social status or gaining notoriety by detesting others, acting out of boredom as a form of entertainment or just the kick for such behavior without fear of consequences [2]. Much of this concerns the anonymity provided by online platforms, which has allowed users to remain unidentified and stay clear of responsibility. Other factors that could trigger motivation for cyberbullying may be peer pressure, social status, and control and power [20]. Anonymity on the internet is a significant facilitator since perpetrators can engage in abusive behavior without being penalized. Additional factors like the need for power and control, revengeful attitudes, and feelings of jealousy or envy may also motivate people to cyberbully others.

Consequences of Cyberbullying

Consequences of cyberbullying and victimization in India: Studies found that bullying among school-going adolescents does occur in India, with some risk factors for bullying and victimization being specific to the Indian context. In India, bullying leads to dire consequences for both the aggressor and the victim. Thus, the impact of cyberbullying on the victim may be huge, from the psychological to the physiological level [10]. The psychological effects (Table 3) include depression and anxiety, low self-esteem and feelings of worthlessness, emotional distress and feelings of powerlessness, social withdrawal, and isolation, academic problems, including decreased concentration and lower grades, fear and paranoia about online interactions, suicidal thoughts, increased tendency for self-harm and suicidal behavior attempts [10]. Physiological effects (Table 4) include sleep disturbances, including insomnia or excessive sleeping; changes in eating habits, which can lead to weight loss or gain; somatic symptoms such as headaches, stomachaches, and muscle tension; increased stress levels and exacerbation of existing health conditions due to stress and emotional turmoil [2,10]. The review suggested that caution has to be exercised while interpreting the results of many studies on bullying from India, mainly because of data collection, instrumentation, and reporting issues. It called for cross-cultural comparisons for prevalence estimates and longitudinal studies to understand the dynamics of bullying and its effects in India [5]. Bullying and victimization were widespread issues among adolescents in India, with the prevalence and characteristics of these issues varying across the different regions of the vast country [5]. 

**Table 3:** Common psychological problems due to cyberbullying among adolescents in India

Psychological problems due to cyberbullying among adolescents in India
Depression
Anxiety
Low self-esteem
Feelings of worthlessness
Emotional distress
Feelings of powerlessness
Social withdrawal
Isolation
Academic problems
Including decreased concentration
Lower grades
Fear and paranoia about online interactions
Suicidal thoughts
Increased tendency for self-harm
Suicidal behavior attempts

**Table 4:** Common physiological problems due to cyberbullying among adolescents in India

Physiological problems due to cyberbullying among adolescents in India
Sleep disturbances including insomnia or excessive sleeping
Changes in eating habits which can lead to weight loss or gain
Somatic symptoms such as headaches, stomachaches, and muscle tension
Increased stress levels
Exacerbation of existing health conditions due to stress and emotional turmoil

The effects of cyberbullying on victims can be detrimental and can reach the point of mental health issues such as depression, anxiety, low self-esteem, and suicidal ideation [10]. This is made worse by the very nature of cyberbullying; there is always a possibility that it will be shared and amplified rapidly online, thereby causing greater trauma to the victim. Moreover, the pernicious effects of cyberbullying spill beyond the individual in terms of their social well-being and performance in academics.

Cyberbullying can result in some devastating consequences for the victim: anxiety, depression, low self-esteem, and even suicide [2]. Additionally, they often face difficulties in academics, withdrawing from social situations, and avoiding technology, actions that will go on to prevent them from developing fully as a person and professional later in life. The effects of cyberbullying also spread much further than the victims themselves and can be seen within communities, families, and the wider social order.

Effect of cyberbullying on mental health: Cyberbullying has very serious effects on mental health. Various mental health problems include depression, anxiety, psychological distress, and post-traumatic stress symptoms. The traumatization of cyberbullying victims can contribute to depressive symptoms, and insomnia, and even result in counterproductive work behavior. Furthermore, the victims often have lower levels of job satisfaction, lower engagement, and higher attrition intentions. For this vulnerable group of students, some of the consequences of cyberbullying include an increase in absenteeism, failure to pay attention, shame and guilt feelings, and anti-social behaviors. Some research also points out those female respondents who were being bullied in cyberspace have developed a higher rate of suicidal ideation compared to males. Cyberbullying has also been linked to appearance anxiety, which, combined with the bullying experience, can exacerbate social anxiety in the victims [15].

Interventions and Coping Strategies

Interventions for cyberbullying and mental health in India: A multi-faceted approach involving stakeholders at various levels is necessary in order to curb this rising menace of cyberbullying and its impact on mental health. The Indian government has made a few efforts through the National Cyber Crime Reporting Portal and the National Cyber Crime Training Centre to upgrade reporting and probe facilities for cybercrime cases. Schools and educational institutions have implemented anti-bullying policies, awareness programs, and counseling services to support victims. However, further efforts are needed to establish a sound legal framework, improve digital literacy, and closer coordination between law enforcement agencies, technology companies, and mental health professionals. Prevention requires the incorporation of cyberbullying prevention into the curriculum, empowering parents to monitor activities, and developing an effective reporting and intervention mechanism [2].

Studies on coping strategies for cyberbullying: These findings reveal the importance of developing and promoting effective coping strategies to mitigate the negative psychological consequences of cyberbullying [21]. Coping behavior helps to act as a mediating factor that helps in reducing the feelings of loneliness that are associated with being a victim of cyberbullying. When cyberbullying victimization is associated with active coping skills, such as seeking help from friends, family, or professionals, the negative feelings that arise from this kind of victimization can be reduced together with psychological impacts [22]. Emotional intelligence is found to alleviate the negative effects of cyberbullying on loneliness. [22]. Also, Gupta et al. reported that the common coping mechanisms among college students were digital advice, technical coping, assertiveness, close support, self-blame, helplessness, retaliation, ignoring, etc. [21].

 These findings underscore the importance of equipping adolescents and youth with the necessary coping skills and emotional intelligence to navigate the challenges posed by cyberbullying. Coping strategies play a critical role in helping adolescents manage the emotional and mental effects of cyberbullying victimization. These strategies could reduce feelings of loneliness in adolescents and give them practical ways to deal with the hurt of being bullied. Seeking social support and engaging in active coping behaviors can make their way through the adverse experiences better, thus making the psychological effect of such incidents more negligible. Emotional intelligence adds to the removal of these bad effects by facilitating adolescents to process and manage their emotions in a way that is much healthier [22].

Research on deep learning techniques to prevent cyberbullying: Deep learning, being a subset of machine learning, has become very instrumental in detecting and mitigating cyberbullying on social media sites. Deep learning models bring a lot of promise toward solving this growing problem by maintaining high accuracy in detecting and classifying cyberbullying content over social media [23]. Several researchers have been developing applications for detecting cyberbullying, using a combination of machine learning and deep learning techniques [23]. These studies have shown promising results in correctly identifying cyberbullying cases and providing timely interventions to prevent further harm [23]. The use of machine learning and deep learning techniques has shown potential in effectively recognizing and preventing cases related to cyberbullying [23]. A review on deep-learning-based cyberbullying detection indicated that it was necessary to focus on this approach since deep-learning models have surpassed the performance of traditional machine-learning models [24]. The authors pointed out that very few comprehensive surveys on the application of deep learning for cyberbullying detection are available, as most surveys available today are quite outdated [24]. A study examined existing literature on deep-learning-based cyberbullying detection and pointed out that further research is required because deep-learning models outperform traditional machine-learning models [25]. Another study proposed a framework for deep learning cyberbully detection on social media, achieving high accuracy in abusive content identification [26]. The researchers applied various feature engineering techniques, including sentiment analysis, linguistic features, and network-based features to construct a robust model capable of detecting instances of cyberbullying. Relating to "ProTect: a hybrid deep learning model for proactive detection" with an enhanced decision tree classifier and Deep Neural Network combined, along with self-attention models using word embeddings of GloVe, to tackle challenges such as issues of overfitting [27]. Further, even sophisticated methods such as transformer networks, including sentence-Destil BERT, were investigated and discussed with respect to binary classification of bullying behavior. Methods that have been applied here include text mining, machine learning models, natural language processing, and social network analysis, among others, which are aimed at giving timely intervention when detected bullying behaviors, ensuring efficient, accurate automated systems are put into effect to enable victims to receive support intensively [28]. The results of such a research endeavor point to great potential in using deep learning techniques to address the increasing problem of cyberbullying by showing better abilities at pinpointing occurrences of online harassment and allowing for timely intervention.

Efforts to address cyberbullying in India: The Indian government has been at the forefront in trying to combat this menace by providing helplines, complaint portals, and provisions in law to stem the tide of online offenses. The Information Technology (IT) Act, 2000, and the Protection of Children from Sexual Offences (POCSO) Act, 2012 have been amended to include provisions related to cyberbullying and online harassment. Apart from these legal measures, various non-governmental organizations and advocacy groups have launched campaigns and educational programs to empower children, parents, and teachers to use the digital world safely [2].

Recommended measures to combat cyberbullying in India: The measures include: (i) Strengthening legal frameworks: Enhancing the legal framework to address cyberbullying is crucial in establishing clear consequences for perpetrators. This includes updating existing laws and regulations to encompass cyberbullying, thereby ensuring that offenders are held accountable for their actions. Moreover, facilitating the swift and efficient resolution of cyberbullying cases through the legal system is paramount in providing justice for victims [2,8,15]; (ii) Promoting digital literacy and cyber safety education: In order to prevent cyberbullying, it is essential to prioritize digital literacy and cyber safety education, especially among children, adolescents, and university students. Implementing comprehensive educational programs that underscore responsible and ethical online behavior can help instill a culture of empathy and digital responsibility. By equipping individuals with the necessary skills to navigate the digital landscape safely, we can mitigate the risks associated with cyberbullying [2,8,15]; (iii) Enhancing reporting and support mechanisms: Efficient reporting mechanisms and support systems for victims of cyberbullying are imperative to provide swift intervention and assistance. Establishing accessible and confidential reporting channels, coupled with dedicated support services, can empower victims to seek help and recourse. Additionally, fostering a supportive and empathetic environment within educational institutions and online platforms can offer crucial emotional and psychological support to those affected by cyberbullying [2,8,15]; (iv) Fostering a culture of empathy and online responsibility: Creating a culture of empathy and online responsibility is fundamental in addressing the root causes of cyberbullying. Encouraging respectful and compassionate interactions in digital spaces, and promoting inclusivity and understanding, can contribute to the prevention of online harassment and abuse. Emphasizing the ethical use of online platforms and advocating for positive digital citizenship can engender a safer and more supportive online environment for all. Combating cyberbullying in India demands a concerted effort and collaboration across governmental bodies, educational institutions, and society as a whole. By prioritizing legal reforms, education, support mechanisms, and fostering a compassionate online culture, we can work towards mitigating the detrimental impact of cyberbullying and creating a safer digital landscape for all individuals [2,8,15].

While these are all good initiatives, comprehensive and coordinated action is further required if effective combating of cyberbullying in India has to be put into practice. According to experts, this calls for a systematic, multi-stakeholder approach with policymakers, law enforcers, educators, mental health professionals, and youth representatives toward developing evidence-based solutions and strengthening the legal and support systems.

Legal and Policy Framework

Laws related to cyberbullying in India: The laws include [2]: (i) Information Technology Act 2000: The ITAct, 2000 (amended in 2008) is a legislation of the Government of India, set up to deal with offenses using the internet or cyber, and the sanction for crimes. This Act defines the crime of cybercrime and penalty for the offense. Cyberbullying is one such offense that affects the person concerned and is hard to survive; the casualty might end it all in extreme cases. It is hard to believe that there is no particular law in India governing cyberbullying, but it is real, and with cyberbullying constantly on the rise in India, this is a frightening fact. The crime of cyberstalking was included in the resolution as a crime in 2013, even though cyberbullying has still not been included. Nevertheless, provisions in Section XI of the rule could allow for aid in cyberbullying activities unless certain exceptional circumstances occur (Table 5).

**Table 5:** Laws related to cyberbullying in India

Law and section	Description
Indian Penal Code of 1860	
Section 503	Sending threatening messages through email
Section 509	Word, gesture or act intended to insult the modesty of a woman
Section 499	Sending defamatory messages through email
Section 500	Email Abuse
Information Technology Act, 2000	
Section 66A	Sending offensive messages through communication services (though this section was struck down by the Supreme Court in 2015 for being unconstitutional)
Section 66D	Cheating by personation by using the computer resource
Section 66E	Violation of privacy
Section 67B	Punishment for publishing or transmitting of material depicting children in sexually explicit acts, in electronic form
Section 72	Breach of confidentiality and privacy
Protection of Children from Sexual Offences Act of 2012 (POCSO)	Protects children below 18 years from sexual harassment, including sexual cyberbullying

Despite these provisions, the laws are not always seen as fully equipped to handle the specific challenges of cyberbullying, and there have been calls for more specific legislation to address this issue

Addressing the challenge of cyberbullying in India (initiatives and recommendations): Various initiatives have been taken in India to address the challenge of cyberbullying. The Ministry of Human Resources has directed schools and colleges to form Anti-Ragging Committees to address cyberbullying [2]. The University Grants Commission has also issued regulations specifically focused on curbing the menace of ragging, which includes provisions to tackle cyberbullying. The National Council of Education Research and Training has also issued guidelines that define the roles of teachers, parents, and students in promoting ethical internet use and reporting online bullying immediately. In addition, it is recommended that educational institutions use built-in filters to prevent harassment by cyberbullies [2].

The Indian government has taken some steps to address the challenge of cyberbullying, such as the establishment of legal provisions, helplines, and awareness campaigns. One of the key initiatives is the IT Act of 2000, which includes provisions for addressing various forms of cybercrime, including harassment and defamation. Additionally, the government has set up reporting portals and helplines to assist victims of cyberbullying and other online crimes. The IT Act, 2000 is the primary legal instrument that deals with prosecuting cybercrimes. This Act, when read with the Indian Penal Code addresses elements of cybercrimes while the Protection of Children from Sexual Offences (POCSO) Act, 2012, addresses cyber sexual offences concerning minors. Besides reporting the incident on the concerned website, a complaint of cyberbullying against a child can be filed in National Cyber Crime Portal, POCSO e-Box, National Commission for Protection of Child Rights (NCPCR) eBaalnidan Portal, Local Police or Cyber Police, Childline 1098. While these efforts are commendable, their impact has been limited due to a lack of awareness and the challenges in enforcing the law. To address the challenge of cyberbullying effectively, a more holistic and collaborative approach is needed [2].

Promoting Internet safety and responsible digital citizenship: Recommended strategies on raising awareness on cyberbullying and ensuring a safe online environment for adolescents: Recommendations for parents and educators. Multi-faceted approaches are needed to address this, which must bring together parents, educators, policymakers, and the tech industry under one roof. Parents and educators should sensitize adolescents about online safety and foster open communication. Policymakers have to enact and enforce laws that delve into cyberbullying, and the tech industry should work on developing tools and algorithms that detect and prevent online harassment.

Creating awareness of cyberbullying and promoting a safe online environment for adolescents involves an all-inclusive approach. Schools and parents need to inform children about digital safety and online etiquette, bullying awareness in general, and the worst consequences their online activities could bring to their lives. An open avenue of communication where children do not have to fear reprimand or judgment for talking about their experiences should be encouraged. Empathy, respect, and responsible online behavior through social-emotional learning programs should also be encouraged. Parents should be involved and, at the same time, respect the privacy of their child in knowing what goes on in the online social dynamics. Schools should design clear policies against cyberbullying and put up reporting mechanisms in the case of students who have been bullied or have witnessed bullying. Spreading awareness about legal frameworks and redressal mechanisms that may provide relief for cyberbullying among students and parents establishing support systems consisting of counselors, peer support groups, and access to mental health resources to help victims of cyberbullying deal with their problems. Educators and caregivers need training on the signs of bullying, when to provide support, and how, and what action to take if cyberbullying is disclosed to them. Schools and communities should launch and sustain initiatives and campaigns against cyberbullying and build the resilience of students. Children need to have a safe and confidential space to report bullying incidents and have it taken seriously, knowing that their problems will be taken care of. When developed, these plans will help deal with cyberbullying on a proactive note and lead to a better and safer environment for adolescents, online and offline.

Limitations

The details presented in this study are based solely on the literature review, which implies that the writers did not gather any original data but rather presented the information in a more critical way. The number of studies included in this research can also be presented as a limitation of this research. Many aspects of cyberbullying are still not well-researched and there is a need for further research in this area. There is a need to develop a universal definition and have methodologically sound research in this field, which can help formulate policies. Research should also focus on the long‑term mental health implications of being a victim and a perpetrator of cyberbullying. Future research should incorporate techniques such as machine learning and artificial intelligence to understand the epidemiology, be able to detect the victims and perpetrators, and improve surveillance for cyberbullying.

## Conclusions

The present study identified that the prevalence of cyberbullying among adolescents in India ranges from 3.3 to 60.56%. This variability in the prevalence and incidence of cyberbullying is due to several factors such as differences in the age, location of samples, methodology used, and different scales used. reporting time frames etc. The review also identified common methods of cyberbullying among adolescents in India as stalking, rumor spreading, making insulting remarks, fun-making, sending hurtful messages, leaking and sharing photos, or videos through electronic media, sharing confidential information with others without the consent of the victim, online harassment, name-calling, trolling, etc. Common psychological issues identified with cyberbullying of adolescents in India were depression, anxiety, low self-esteem, feelings of worthlessness, emotional distress, feelings of powerlessness, etc.

Cyberbullying is becoming one of the major concerns for many adolescents, which is causing unexpected deviances and negative outcomes in their domains of life. Cyberbullying is not going to disappear soon. Rather, it will manifold itself by many times due to rapid digitalization. Cyberbullying has the potential to have a tremendous adverse effect on the mental health of the victims. The high prevalence of cyberbullying among adolescents and university students in India calls for an urgent and whole-hearted response. While the Government of India has brought in legal provisions, reporting portals, and even awareness campaigns to combat cyberbullying, much more needs to be done to deal with such a multi-faceted problem effectively. Future research should familiarize itself with the exact prevalence rates and work on the enhancement of digital literacy, reporting mechanisms, and support systems, thereby giving birth to a culture of empathy and responsibility in online conduct. Governmental bodies, organizations, and academic institutions should come together to devise comprehensive strategies for the prevention of cyberbullying and support of victims. Further research studies on bullying and victimization should be conducted in India, providing more valid and reliable data that will aid in the fight for a safe online environment for young people. Therefore, it is necessary to make all the stakeholders aware of this menace. Efforts should be made to make all children and adolescents aware of the legal provisions and how indulging in cyberbullying can land them in conflict with the law. The victims of cyberbullying should be enabled to report the same at the earliest and they should be educated as to how to protect themselves from further cyberbullying. The parents and teachers should focus on certain behaviors that may be indicative of being a victim or a perpetrator in cyberspace. Parents and teachers should inculcate safe behavior in cyberspace within children and adolescents. All efforts must be taken for successful avoidance and response to it, alongside providing the children and adolescents with tools to lessen their own risk of victimization. The removal of cyberbullying risks is going to require a coordinated and collaborative effort on the part of diverse youth advocates.

## References

[1] Ahmed JO (2023). Social media psychology and mental health. Middle East Curr Psychiatr.

[2] Kaur M, Saini M (2023). Indian government initiatives on cyberbullying: a case study on cyberbullying in Indian higher education institutions. Educ Inf Technol (Dordr).

[3] Shoib S, Philip S, Bista S (2022). Cyber victimization during the COVID-19 pandemic: a syndemic looming large. Health Sci Rep.

[4] Saif AN, Purbasha AE (2023). Cyberbullying among youth in developing countries: a qualitative systematic review with bibliometric analysis. Child Youth Serv Rev.

[5] Thakkar N, Van Geel M, Vedder P (2021). A systematic review of bullying and victimization among adolescents in India. Int J Bullying Prev.

[6] Khalaf AM, Alubied AA, Khalaf AM, Rifaey AA (2023). The impact of social media on the mental health of adolescents and young adults: a systematic review. Cureus.

[7] Chun J, Lee J, Kim J, Lee S (2020). An international systematic review of cyberbullying measurements. Comp Human Behav.

[8] Bashir Shaikh F, Rehman M, Amin A (2020). Cyberbullying: a systematic literature review to identify the factors impelling university students towards cyberbullying. IEEE Access.

[9] Gustafsson Gustafsson, E E (2017). Gender differences in cyberbullying victimization among adolescents in Europe. A systematic review. Malmö University: Faculty of health and society, Department of criminology. Gender Differences in Cyberbullying Victimization Among Adolescents in Europe. A Systematic Review.

[10] Ranjith PJ, Vranda MN, Kishore MT (2023). Predictors, prevalence, and patterns of cyberbullying among school-going children and adolescents. Indian J Psychiatry.

[11] Sharma D, Kishore J, Sharma N, Duggal M (2017). Aggression in schools: cyberbullying and gender issues. Asian J Psychiatr.

[12] Kodapally B, Mathews E, Kodali PB, Thankappan KR (2021). Bullying victimization and its associated factors among adolescents in Kozhikode district, Kerala, India: a mixed-methods study [version 1; peer review: 1 approved]. Wellcome Open Res.

[13] Kaur D, Vashist S, Negi H, Khan A, Jeganathan J, Nandini M (2024). Prevalence and level of cyber bullying among nursing students at selected nursing college, Dehradun, Uttarakhand, india. Educ Admin Theory Pract.

[14] Sathyanarayana Rao TS, Bansal D, Chandran S (2018). Cyberbullying: a virtual offense with real consequences. Indian J Psychiatry.

[15] Bansal S, Garg N, Singh J, Van Der Walt F (2023). Cyberbullying and mental health: past, present and future. Front Psychol.

[16] Vranda MN, Doraiswamy P, Prabhu JR, Ajayan A, Priyankadevi S (2023). Content analysis of cyberbullying coverage in newspapers- a study from Bengaluru, India. Ind Psychiatry J.

[17] (2024). 85% Indian kids have experienced cyberbullying, highest in the world, finds new survey. https://theprint.in/india/85-indian-kids-have-experienced-cyberbullying-highest-in-the-world-finds-new-survey/1074175/..

[18] Maji S, Abhiram AH (2023). "Mental health cost of internet’’: a mixed-method study of cyberbullying among Indian sexual minorities. Telemat Info Rep.

[19] Oblad T (2021). A holistic overview of cyberbullying across the world: review of theories and models. Anxiety Disorders - The New Achievements.

[20] Kowalski RM, Giumetti GW, Schroeder AN, Lattanner MR (2014). Bullying in the digital age: a critical review and meta-analysis of cyberbullying research among youth. Psychol Bull.

[21] Gupta S, Soohinda G, Sampath H, Dutta S (2023). Cyberbullying: a study of its extent, coping resources, and psychological impact among college students. Ind Psychiatry J.

[22] Maurya C, Muhammad T, Dhillon P, Maurya P (2022). The effects of cyberbullying victimization on depression and suicidal ideation among adolescents and young adults: a three year cohort study from India. BMC Psychiatry.

[23] Raj M, Singh S, Solanki K, Selvanambi R (2022). An application to detect cyberbullying using machine learning and deep learning techniques. SN Comput Sci.

[24] Hasan MdT, Hossain MdAE, Mukta MdSH, Akter A, Ahmed M, Islam S (2023). A review on deep-learning-based cyberbullying detection. Future Internet.

[25] Roy R, Mukherjee S, Chaturvedi M, Agarwal K, Kannan AT (2014). Prevalence and predictors of psychological distress among school students in Delhi. J Indian Assoc Child Adolescent Ment Health.

[26] S N, M S, Chandrasekaran S, K M, Singh Pundir AK, R S, Lingaiah TB (2022). Deep learning approaches for cyberbullying detection and classification on social media. Comput Intell Neurosci.

[27] Nitya Harshitha T, Prabu M, Suganya E, Sountharrajan S, Bavirisetti DP, Gadde N, Uppu LS (2024). ProTect: a hybrid deep learning model for proactive detection of cyberbullying on social media. Front Artif Intell.

[28] Yadav J, Kumar D, Chauhan D (2020). Cyberbullying detection using pre-trained BERT model. International Conference on Electronics and Sustainable Communication Systems (ICESC).

